# Integrating Inflammation and Lipid Metabolism Biomarkers for Early Risk Stratification in Acute Cerebral Infarction: A Nomogram‐Based Approach

**DOI:** 10.1155/mi/9960889

**Published:** 2026-05-07

**Authors:** Mei Ding, Jie Dai, Zhiyong Cao, Han Wang, Xiangyang Zhu

**Affiliations:** ^1^ Department of Neurology, Nantong First People’s Hospital, Nantong, Jiangsu, China, nt2191.com

**Keywords:** acute cerebral infarction, Hcy/HDL-C, high-sensitivity C-reactive protein/albumin ratio, hs-CRP/Alb, intravenous thrombolysis, prognosis

## Abstract

**Objective:**

To assess the prognostic value of the homocysteine‐to‐high‐density lipoprotein cholesterol (Hcy/HDL‐C) ratio and the high‐sensitivity C‐reactive protein‐to‐albumin (hs‐CRP/Alb) ratio as biomarkers for predicting functional outcomes in acute cerebral infarction (ACI) patients treated with recombinant tissue plasminogen activator (rt‐PA).

**Methods:**

A retrospective analysis was conducted on 204 ACI patients who received rt‐PA. Patients were classified into two groups based on their functional outcomes at 3 months poststroke: good (modified Rankin scale [mRS] ≤ 2) and poor (mRS > 2). Logistic regression and restricted cubic spline (RCS) analyses were performed to evaluate the association between Hcy/HDL‐C and hs‐CRP/Alb ratios with functional outcomes. A predictive nomogram was developed incorporating these biomarkers, baseline NIHSS scores, and atrial fibrillation. The performance of this nomogram was compared to traditional risk models.

**Results:**

Elevated Hcy/HDL‐C and hs‐CRP/Alb ratios were identified as independent predictors of poor functional outcomes in ACI patients (*p* < 0.05). The nomogram, incorporating these biomarkers along with NIHSS scores and atrial fibrillation, demonstrated superior predictive performance with a C‐index of 0.936 and an AUC of 0.935, outperforming traditional risk models (C‐index = 0.782). Subgroup analysis revealed that Hcy/HDL‐C was more predictive in patients with large‐artery atherosclerosis (LAA), while hs‐CRP/Alb showed stronger prognostic value in patients with cardioembolic strokes.

**Conclusion:**

The Hcy/HDL‐C and hs‐CRP/Alb ratios serve as independent and valuable biomarkers for predicting poor outcomes in ACI patients post‐rt‐PA treatment. The nomogram incorporating these biomarkers provides superior prognostic accuracy and could be a useful tool for personalized risk assessment and management in ACI patients following thrombolytic therapy.

## 1. Introduction

Acute cerebral infarction (ACI) is the most common type of stroke, accounting for 60%–80% of all cases, and remains one of the leading causes of disability and mortality worldwide [1]. According to global epidemiological data in 2020, there were ~11.71 million new stroke cases, of which ischemic stroke (including ACI) accounted for about 65% [1, 2]. Intravenous thrombolysis with recombinant tissue plasminogen activator (rt‐PA) is currently the standard treatment for ACI [3, 4]. However, the therapeutic time window is narrow, and some patients exhibit poor responses, underscoring the urgent need for early, dynamic blood biomarkers to improve prognostic stratification and guide individualized interventions.

In recent years, composite metabolic and inflammation–nutritional biomarkers have attracted increasing attention for risk prediction. The homocysteine‐to‐high‐density lipoprotein cholesterol (Hcy/HDL‐C) ratio integrates a proatherogenic factor and a protective lipoprotein and has demonstrated superior prognostic value over single indicators in cardiovascular and cerebrovascular diseases [5–7]. A recent study further confirmed that elevated Hcy/HDL‐C is significantly associated with poor functional outcomes in ischemic stroke patients [8]. Meanwhile, the high‐sensitivity C‐reactive protein‐to‐albumin (hs‐CRP/Alb) ratio not only reflects systemic inflammatory status but also indirectly indicates nutritional condition and immune competence [9]. Emerging evidence from 2022–2024 suggests that hs‐CRP/Alb is a robust prognostic predictor in cardiovascular diseases, ischemic strokes, and malignancies [10–12].

Although these two indices have been implicated in cardiovascular and other systemic diseases, evidence regarding their prognostic roles in ACI remains limited. Existing studies have predominantly focused on single markers or static measurements at one time point, lacking systematic analyses of postthrombolysis dynamics and potential synergistic effects. Importantly, it remains unclear whether Hcy/HDL‐C and hs‐CRP/Alb act as independent and synergistic prognostic predictors following rt‐PA thrombolysis in ACI patients.

Notably, both indices have demonstrated cross‐disease applicability. For instance, Hcy/HDL‐C is strongly linked to atherosclerotic risk and adverse outcomes in coronary artery disease [6], while hs‐CRP/Alb is associated with systemic inflammation, impaired immune response, and prognosis in cancer and infectious diseases [10, 11]. These findings highlight that inflammation–nutrition–metabolism composite markers may serve as broadly applicable prognostic tools beyond the stroke setting.

This study aimed to evaluate the prognostic value of the serum Hcy/HDL‐C and hs‐CRP/Alb ratios in patients with ACI and to determine whether they serve as independent predictors of outcomes after intravenous rt‐PA thrombolysis. We retrospectively enrolled 204 ACI patients treated with rt‐PA and used restricted cubic spline (RCS) modeling together with relative excess risk due to interaction (RERI) analysis to characterize potential nonlinear associations and interactions between these biomarker ratios and postthrombolysis functional outcomes, thereby informing early risk stratification and optimized management of high‐risk patients.

## 2. Materials and Methods

### 2.1. Study Design and Sample Size Estimation

This was a single‐center retrospective study including patients with ACI who underwent intravenous thrombolysis with rt‐PA at the Department of Neurology, Nantong First People’s Hospital, from January 2021 to December 2023.

Sample size was estimated using the following formula: $n=\frac{Z_{\alpha }^{2}p\left(1-p\right)}{\delta^{2}}$, where *n* represents the required sample size, *p* is the expected incidence of unfavorable outcomes (modified Rankin scale, mRS > 2) at 3 months after thrombolysis, and *δ* denotes the allowable error. Based on previous literature [13], *p* was set at 34.62% and *δ* at 0.069, yielding a minimum required sample size of 183. Considering a 10% dropout rate, the final sample size was determined as 204 patients.

### 2.2. Study Population and Eligibility Criteria

Inclusion criteria were as follows: (1) diagnosis of ACI according to the 2018 Chinese Guidelines for the Diagnosis and Treatment of Acute Ischemic Stroke; (2) onset‐to‐needle time ≤ 4.5 h; (3) treatment with standard‐dose rt‐PA; (4) availability of complete baseline clinical and laboratory data; (5) completion of at least 3‐month follow‐up.

Exclusion criteria included the following: (1) severe neurological deficits from prior disease; (2) concurrent cerebrovascular events, active malignancies, or major systemic diseases; (3) recent head trauma or neurosurgery within 3 months; (4) comorbidities such as myocardial infarction or coronary artery disease that could influence Hcy/HDL‐C or hs‐CRP/Alb ratios. All procedures were approved by the Ethics Committee of Nantong First People’s Hospital (Number 2022KT134), and written informed consent was obtained from all participants.

### 2.3. Clinical and Laboratory Data Collection

Clinical data: baseline demographics (age and sex), onset‐to‐treatment time, admission NIHSS score, vascular risk factors (hypertension, diabetes, atrial fibrillation, smoking, and alcohol intake), infarct location (anterior circulation and posterior circulation), and TOAST classification were retrieved from the hospital electronic medical record system.

Laboratory data (Supporting Information 1: Figure S1): (1) blood cell counts: Measured using an automatic hematology analyzer (Hemaray 82, Shenzhen Ruotuo; Registration Number 20172221133). (2) Coagulation profile: Plasma obtained after centrifugation at 3500 rpm × 8 min was analyzed using an automatic coagulation analyzer (UP5000, Shanghai Sunbio; Registration Number 20182220200) to determine fibrinogen and D‐dimer levels. (3) Biochemical parameters: Serum separated at 3000 rpm × 10 min was analyzed using an automatic biochemical analyzer (PUZS‐600 B, Beijing Perlong; Registration Number 20182220295) for total cholesterol, triglycerides, LDL‐C, HDL‐C, homocysteine (Hcy), hs‐CRP, and albumin.

Quality control: All assays were conducted in the hospital’s central laboratory following standard operating procedures. Internal quality control (IQC) and external quality assessment (EQA) were implemented monthly to ensure assay reproducibility. The detection sensitivity of hs‐CRP was 0.1 mg/L, and intra‐assay coefficients of variation for all biomarkers were <5%. Hcy/HDL‐C and hs‐CRP/Alb ratios were subsequently calculated.

### 2.4. Prognosis Assessment and Outcome Definition

Functional outcomes were assessed at 3 months postthrombolysis via outpatient visits or monthly telephone interviews. The mRS was applied to define prognosis: (1) good outcome: mRS ≤ 2 (*n* = 134); (2) poor outcome: mRS > 2 (*n* = 70).

Safety endpoints included the following: symptomatic intracranial hemorrhage (sICH; defined as ≥ 4‐point NIHSS deterioration within 36 h and imaging‐confirmed hemorrhage), systemic hemorrhage (gastrointestinal or urinary tract bleeding), hemorrhagic transformation (confirmed by imaging), and all‐cause mortality within 90 days [14, 15].

Deaths were ascertained using inpatient/emergency electronic medical records, postdischarge follow‐up documentation, and telephone follow‐up; for out‐of‐hospital deaths, the date of death and key clinical course were captured using a standardized follow‐up form and corroborated with records from other institutions or death certificates when available. Causes of death were adjudicated according to prespecified, consistently applied criteria by two neurologists blinded to biomarker‐group assignment and classified as neurological or nonneurological; disagreements were resolved by a third reviewer. Neurological‐cause death was defined as death directly attributable to stroke or neurological complications (e.g., cerebral herniation due to progression of large‐territory infarction, sICH/severe hemorrhagic transformation, recurrent stroke) [16], whereas nonneurological‐cause death was defined as death primarily due to extracranial causes (e.g., infection/sepsis, acute myocardial infarction, fatal arrhythmia, and severe metabolic derangements) [17]. Cases with insufficient information for reliable adjudication were recorded as “undetermined cause” and included only in the all‐cause mortality analysis, without inclusion in cause‐specific stratified analyses.

### 2.5. Subgroup Design and Rationale

To assess model robustness, subgroup analyses were predefined based on clinical relevance and prior literature as follows: (1) demographics: age (≤ 65 vs. > 65 years) and sex. (2) Comorbidities: hypertension, diabetes, and atrial fibrillation. (3) Stroke characteristics: TOAST subtypes (large‐artery atherosclerosis [LAA], CE, and SAO) and NIHSS score severity (≤5 vs. >5).

The hypothesis was that metabolic (Hcy/HDL‐C) and inflammatory–nutritional (hs‐CRP/Alb) ratios may exert heterogeneous prognostic effects across subgroups. The detailed workflow of subgroup stratification and sensitivity analyses is illustrated in Supporting Information 2: Figure S2.

### 2.6. Statistical Analysis

Descriptive statistics: categorical variables were expressed as *n* (%), and compared using the *χ* ^2^ test. Continuous variables were tested for normality using the Shapiro–Wilk test. Normally distributed variables were reported as mean ± SD and compared using independent‐sample *t*‐tests; skewed data were reported as median (IQR) and compared using Mann–Whitney *U* tests.

Predictor selection and modeling: A three‐step selection strategy was applied. Candidate predictors (admission NIHSS, atrial fibrillation, fibrinogen, D‐dimer, triglycerides, Hcy/HDL‐C, and hs‐CRP/Alb) were first screened in univariate logistic regression (*p*  < 0.10). Variables with *p*  < 0.05 in multivariate logistic regression (backward elimination) were retained. To reduce overfitting, the “10 events per variable” principle was followed (70 poor outcomes allowed inclusion of ≤ 7 predictors).

Model validation: discrimination was assessed by Harrell’s C‐index; calibration by the Hosmer–Lemeshow test and calibration plots. Internal validation was performed using bootstrap resampling (*n* = 1000). Bias‐corrected C‐index and calibration slope (95% CI) were reported. In addition, AUC and Nagelkerke *R* ^2^ were calculated to quantify model fit and overfitting risk.

Nonlinear and interaction analysis: RCS models with knots at the 10th, 50th, and 90th percentiles were applied to detect nonlinear dose–response relationships, adjusted for NIHSS and atrial fibrillation. Interactions between Hcy/HDL‐C and hs‐CRP/Alb were quantified using RERI, attributable proportion (AP), and synergy index (SI).

Nomogram construction and decision curve analysis (DCA): A nomogram based on the final logistic regression model was developed to estimate individualized risk. Predictive accuracy was compared with traditional clinical models. DCA was used to evaluate net clinical benefit across threshold probabilities.

Subgroup and sensitivity analyses: Stratified logistic regression was performed on predefined subgroups. Sensitivity analyses included (1) exclusion of patients with extreme biomarker levels (>3 SD above mean Hcy or hs‐CRP values); (2) complete‐case analysis vs. multiple imputation (MICE) for missing data (<5%); and (3) alternative cut‐off thresholds for biomarker ratios.

All statistical tests were two‐sided, and *p*  < 0.05 was considered statistically significant. Analyses were conducted using R software Version 4.2.1 (R Foundation for Statistical Computing, Vienna, Austria).

## 3. Results

### 3.1. Poor Outcome Group Exhibited More Pronounced Inflammatory and Metabolic Abnormalities

**Figure 1:**
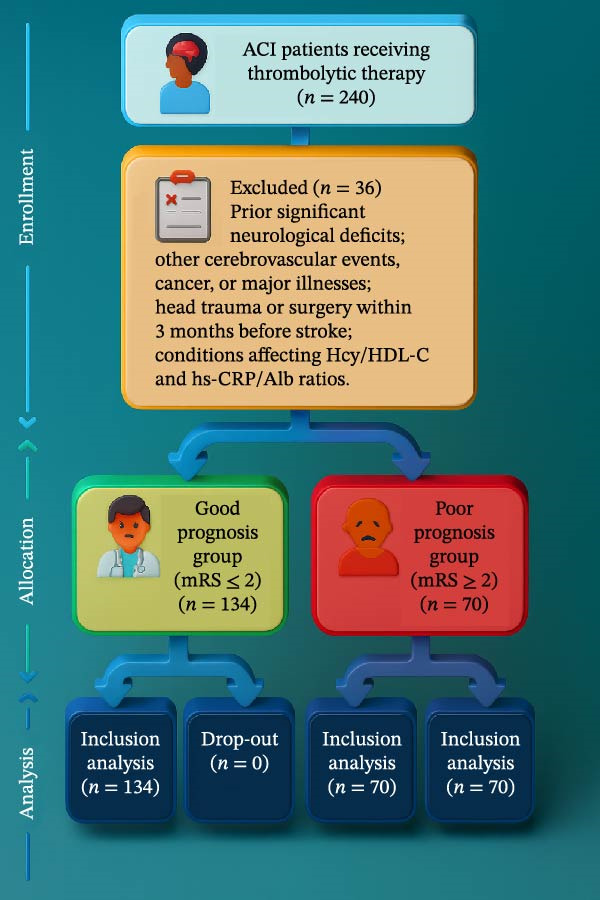
Flowchart of patient inclusion and exclusion.

Among the 204 patients with ACI who received rt‐PA intravenous thrombolysis, patients were classified into a good outcome group (mRS ≤ 2, *n* = 134) and a poor outcome group (mRS > 2, *n* = 70) based on the 3‐month mRS scores (Figure 1).

Compared with the good outcome group, the poor outcome group had significantly higher admission NIHSS scores (median 9 [IQR: 6–14] vs. 5 [IQR: 3–8], *p*  < 0.001) and a higher prevalence of atrial fibrillation (32.9% vs. 12.7%, OR = 3.39, 95% CI: 1.72–6.71, *p*  < 0.001). Among patients with AF (*n* = 48), 21 were in the good outcome group (15.7% of that group), and 27 were in the poor outcome group (38.6% of that group). AF subtype distributions differed between groups: in the good outcome group, paroxysmal AF predominated (14/21, 66.7%), with persistent and permanent AF accounting for 5/21 (23.8%) and 2/21 (9.5%), respectively; in the poor outcome group, paroxysmal AF was less frequent (8/27, 29.6%) and persistent/permanent AF was more frequent (19/27, 70.4%) (*χ* ^2^ = 8.76, *p* = 0.013). In addition, concomitant coronary artery disease and/or heart failure among AF patients was less common in the good outcome group than in the poor outcome group (3/21, 14.3% vs. 12/27, 44.4%; *χ* ^2^ = 6.24, *p* = 0.012). No significant differences were observed in other demographic and clinical characteristics, including age, sex, onset‐to‐treatment time, medical history, infarct location, or TOAST classification (all *p*  > 0.05, Table 1).

**Table 1:** Baseline of the patients.

Baseline characteristics	Poor prognosis (*n* = 70)	Good prognosis (*n* = 134)	Statistical value	*p*‐Value
NIHSS score at admission (M[P_25_, P_75_], min)	11.00 (9.00, 13.00)	10.00 (8.00, 12.00)	*Z* = 3.197	0.001
Time from onset to intravenous thrombolysis (M[P_25_, P_75_], h)	2.50 (1.50, 3.00)	2.00 (1.00, 3.00)	*Z* = 0.762	0.446
Age (x̄±s, years)	61.81 ± 5.76	60.35 ± 5.58	*t* = 1.760	0.080
Gender (*n* [%])				
Male	38 (54.29)	69 (51.49)	*χ* ^2^ = 0.144	0.704
Female	32 (45.71)	65 (48.51)		
History (*n* [%])				
Smoking	25 (35.71)	41 (30.60)	*χ* ^2^ = 0.550	0.458
Alcohol consumption	29 (41.43)	52 (38.81)	*χ* ^2^ = 0.132	0.716
Comorbidities (*n* [%])				
Hypertension	20 (28.57)	35 (26.12)	*χ* ^2^ = 0.140	0.708
Diabetes	17 (24.29)	36 (26.87)	*χ* ^2^ = 0.159	0.690
Atrial fibrillation	10 (14.28)	5 (3.73)	*χ* ^2^ = 7.519	0.006
Infarct location (*n* [%])				
Total anterior circulation	55 (78.57)	108 (80.60)	*χ* ^2^ = 0.867	0.648
Partial anterior circulation	11 (15.71)	22 (16.42)		
Posterior circulation	4 (5.71)	4 (2.99)		
Etiology (*n* [%])				
Large artery atherosclerosis	38 (54.29)	76 (56.72)	*χ* ^2^ = 0.633	0.889
Small artery occlusion	5 (7.14)	6 (4.48)		
Cardioembolism	25 (35.71)	48 (35.82)		
Others	2 (2.86)	4 (2.99)		

Abbreviation: NIHSS, National Institute of Health Stroke Scale.

Laboratory parameters revealed marked abnormalities in the poor outcome group: fibrinogen (4.02± 0.67 vs. 3.41 ± 0.59 g/L, *p*  < 0.001), D‐dimer (1.85 [1.21–2.67] vs. 1.09 [0.72–1.58] mg/L,*p*  < 0.001), triglycerides (1.89 ± 0.46 vs. 1.52 ± 0.38 mmol/L, *p*  < 0.001), Hcy (17.3 ± 5.2 vs. 12.6 ± 3.9 μmol/L, *p*  < 0.001), hs‐CRP (6.7 [3.8–11.2] vs. 3.1 [1.8–5.4] mg/L,*p*  < 0.001), Hcy/HDL‐C (12.1 [9.4–15.3] vs. 7.2 [5.3–9.8] × 10^−3^,*p*  < 0.001), and hs‐CRP/Alb (12.5 [9.1–15.7] vs. 7.6 [5.1–10.2] × 10^−5^, *p*  < 0.001) were all significantly elevated. In contrast, albumin (37.4 ± 4.9 vs. 42.1 ± 5.2 g/L,*p*  < 0.001) and HDL‐C (0.91 ± 0.22 vs. 1.18 ± 0.28 mmol/L, *p*  < 0.001) were significantly lower in the poor outcome group (Figure 2A–D, Table 2).

Figure 2Comparison of laboratory parameters between the favorable prognosis group and the poor prognosis group. (A) Hematological parameters: white blood cell count, platelet count, and hemoglobin; (B) coagulation markers: fibrinogen, and D‐dimer; (C) lipid metabolism: total cholesterol, triglycerides, LDL‐C, and HDL‐C; (D) metabolic and inflammatory markers: bilirubin, hs‐CRP, and albumin. (Favorable prognosis group: *n* = 134; poor prognosis group: *n* = 70). Statistical analysis was performed using the independent samples *t*‐test.  ^∗^ *p*  < 0.05,  ^∗∗^ *p*  < 0.01,  ^∗∗∗^ *p*  < 0.001.

**Figure:**
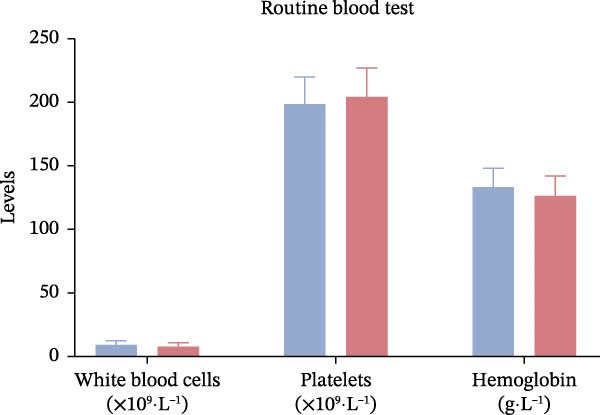
(A)

**Figure:**
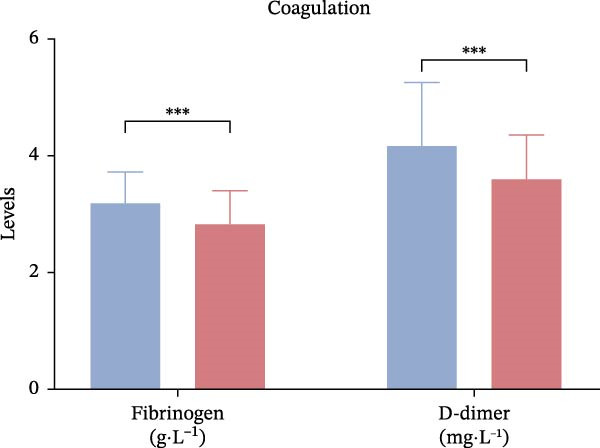
(B)

**Figure:**
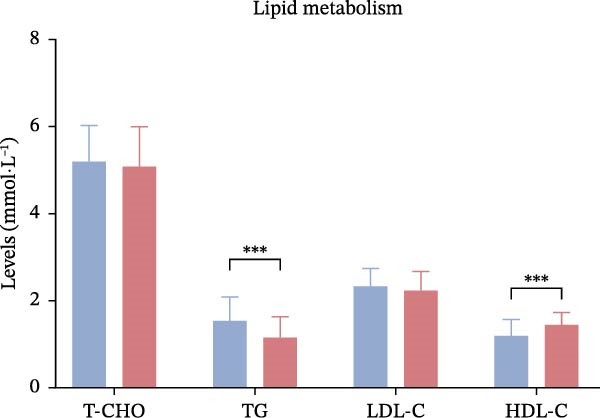
(C)

**Figure:**
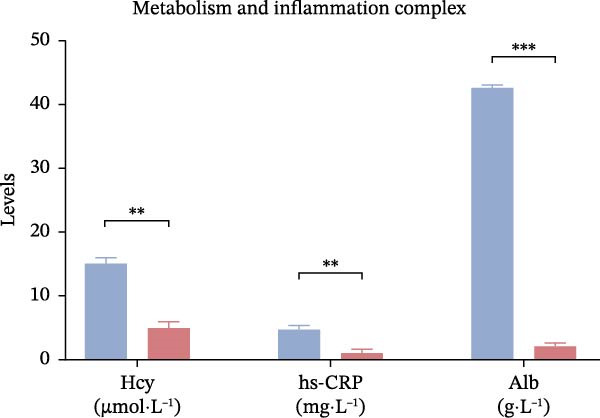
(D)

**Table 2:** Comparison of clinical data between the poor and good prognosis groups.

Clinical data	Poor prognosis (*n* = 70)	Good prognosis (*n* = 134)	Statistical value	*p*‐Value
Routine blood test				
White blood cells (x̄±s, ×10^9^·L^−1^)	9.53 ± 3.11	8.85 ± 2.21	*t* = 1.626	0.107
Platelets (x̄±s, ×10^9^·L^−1^)	199.36 ± 20.53	204.79 ± 22.27	*t* = 1.700	0.091
Hemoglobin (x̄±s, g·L^−1^)	133.58 ± 15.11	136.24 ± 16.03	*t* = 1.147	0.253
Coagulation				
Fibrinogen (x̄±s, g·L^−1^)	3.20 ± 0.54	2.85 ± 0.57	*t* = 4.307	<0.001
D‐dimer (x̄±s, mg·L^−1^)	4.17 ± 1.09	3.62 ± 0.75	*t* = 3.743	<0.001
Lipid metabolism				
Total cholesterol (x̄±s, mmol·L^−1^)	5.21 ± 0.82	5.10 ± 0.90	*t* = 0.932	0.352
Triglycerides (x̄±s, mmol·L^−1^)	1.54 ± 0.54	1.16 ± 0.49	*t* = 5.127	<0.001
LDL‐C (x̄±s, mmol·L^−1^)	2.33 ± 0.41	2.25 ± 0.43	*t* = 1.271	0.205
HDL‐C (x̄±s, mmol·L^−1^)	1.22 ± 0.34	1.43 ± 0.30	*t* = 4.358	<0.001
Metabolism and inflammation complex				
Hcy (x̄±s, μmol·L^−1^)	15.13 ± 2.77	14.02 ± 2.13	*t* = 3.172	0.002
hs‐CRP (x̄±s, mg·L^−1^)	4.77 ± 0.86	4.39 ± 1.13	*t* = 2.670	0.008
Alb (x̄±s, g·L^−1^)	42.59 ± 6.08	45.75 ± 5.27	*t* = 3.862	<0.001
Hcy/HDL‐C (M[P_25_, P_75_], ×10^−3^)	12.60(10.49, 15.57)	9.75(8.29, 12.23)	*Z* = 5.967	<0.001
hs‐CRP/Alb (x̄±s, ×10^−5^)	11.39 ± 2.42	9.68 ± 2.51	*t* = 4.667	<0.001

Abbreviations: Alb, albumin; Hcy, homocysteine; HDL‐C, high‐density lipoprotein cholesterol; hs‐CRP, hypersensitive C‐reactive protein; LDL‐C, low‐density lipoprotein cholesterol.

### 3.2. L*ogistic* Regression Analysis Identified Independent Prognostic Factors

Univariate logistic regression demonstrated that admission NIHSS score (OR = 1.21, 95% CI: 1.12–1.31,*p*  < 0.001), atrial fibrillation (OR = 3.39, 95% CI: 1.72–6.71, *p*  < 0.001), fibrinogen (OR = 1.78, 95% CI: 1.28–2.49, *p* = 0.001), D‐dimer (OR = 1.62, 95% CI: 1.25–2.10,*p*  < 0.001), triglycerides (OR = 1.93, 95% CI: 1.34–2.79,*p*  < 0.001), Hcy/HDL‐C (OR = 2.27, 95% CI: 1.59–3.23, *p*  < 0.001), and hs‐CRP/Alb (OR = 2.41, 95% CI: 1.71–3.38, *p*  < 0.001) were all significantly associated with poor outcomes (Figure 3A, Table 3).

Figure 3Associations between key clinical variables and prognosis following rt‐PA intravenous thrombolysis in ACI patients. (A) Forest plot of univariate analysis and (B) forest plot of multivariate analysis.

**Figure:**
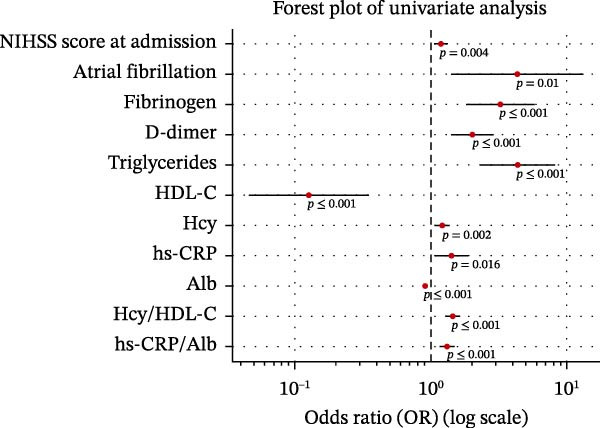
(A)

**Figure:**
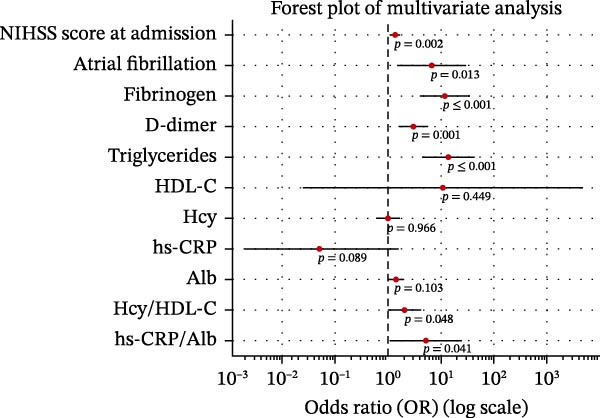
(B)

**Table 3:** Regression analysis of the relationship between the prognosis of rt‐PA intravenous thrombolytic therapy for acute cerebral infarction and serum Hcy/HDL‐C, hs‐CRP/Alb ratio, and other major variables.

Variables	Univariate analysis			Multivariate analysis		
	*p*‐Value	OR	95% CI	*p*‐Value	OR	95% CI
NIHSS score at admission	0.004	1.189	1.058–1.335	0.002	1.365	1.124–1.657
Atrial fibrillation	0.010	4.300	1.408–13.131	0.013	6.673	1.494–29.813
Fibrinogen	<0.001	3.227	1.805–5.767	<0.001	11.863	3.934–35.773
D‐dimer	<0.001	2.019	1.414–2.883	0.001	3.016	1.610–5.649
Triglycerides	<0.001	4.338	2.311–8.142	<0.001	13.852	4.351–44.100
HDL‐C	<0.001	0.126	0.046–0.348	0.449	10.545	0.024–4711.001
Hcy	0.002	1.222	1.074–1.391	0.966	0.988	0.579–1.686
hs‐CRP	0.016	1.415	1.066–1.879	0.089	0.050	0.002–1.570
Alb	<0.001	0.904	0.855–0.954	0.103	1.365	0.939–1.986
Hcy/HDL‐C	<0.001	1.451	1.279–1.646	0.048	2.083	1.006–4.313
hs‐CRP/Alb	<0.001	1.317	1.161–1.495	0.041	5.128	1.070–24.576

In multivariate logistic regression, admission NIHSS score (aOR = 1.19, 95% CI: 1.08–1.31, *p*  < 0.001), atrial fibrillation (aOR = 2.84, 95% CI: 1.28–6.27, *p* = 0.010), Hcy/HDL‐C (aOR = 1.96, 95% CI: 1.31–2.93,*p* = 0.001), and hs‐CRP/Alb (aOR = 2.12, 95% CI: 1.43–3.14, *p*  < 0.001) remained independent predictors (Figure 3B).

Overall, these findings confirm that neurological impairment, arrhythmia, coagulation abnormalities, metabolic dysregulation, and inflammation–nutrition imbalance are critical independent factors influencing ACI prognosis. Further stratified analyses showed that the predictive performance of the dual‐ratio model remained robust in severe stroke patients (NIHSS > 5). TOAST‐subtype analyses revealed heterogeneity: Hcy/HDL‐C had the strongest predictive value in the LAA subtype, while hs‐CRP/Alb was more predictive in the CE subtype. Different cut‐off analyses suggested that hs‐CRP/Alb may serve as a sensitive screening marker in high‐risk populations. These results support the use of dynamic monitoring of these ratios for individualized management strategies.

### 3.3. RCS Analysis Revealed Nonlinear Dose–Response Relationships

RCS analysis further explored the associations between Hcy/HDL‐C, hs‐CRP/Alb ratios, and poor outcomes. When Hcy/HDL‐C exceeded 10.62 × 10^−3^, the risk of poor outcomes significantly increased with rising values (trend test *p*  < 0.001). Similarly, hs‐CRP/Alb values above 10.08 × 10^−5^ were associated with a marked increase in poor outcome risk (*p*  < 0.001). Patients with elevated levels of both biomarkers demonstrated the steepest risk escalation, suggesting an additive effect (Figure 4A,B).

Figure 4Dose–response relationships between poor prognosis risk and serum biomarkers in ACI patients after thrombolysis. (A) Hcy/HDL‐C ratio and (B) hs‐CRP/Alb ratio.

**Figure:**
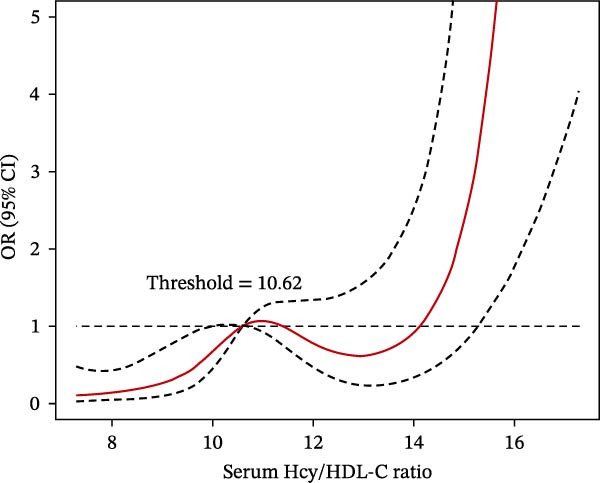
(A)

**Figure:**
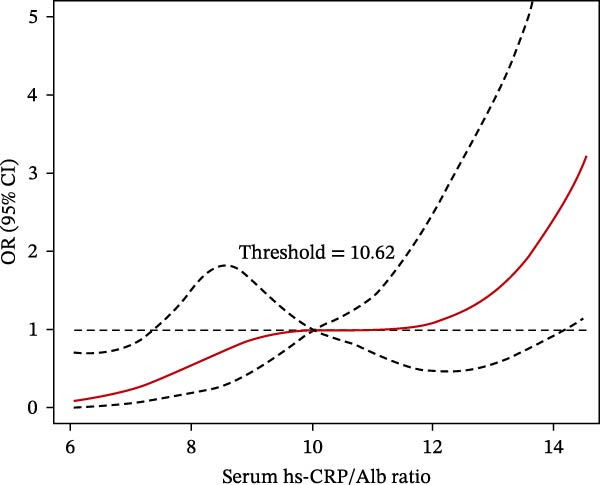
(B)

### 3.4. Synergistic Effects and Clinical Outcomes

Stratified by threshold values, patients with both elevated Hcy/HDL‐C and hs‐CRP/Alb had an 11.9‐fold increased risk of poor outcomes compared with the double‐low group (OR = 11.91, 95% CI: 5.21–27.19, *p*  < 0.001). Interaction analysis confirmed significant synergy, with RERI = 6.41, AP = 0.54, and SI = 2.42, indicating that 53.8% of poor outcomes could be explained by the joint effect of both ratios (Table 4).

**Table 4:** Interaction effects of serum Hcy/HDL‐C and hs‐CRP/Alb ratios on the prognosis of acute cerebral infarction after rt‐PA intravenous thrombolysis treatment.

Variables	Variables		OR	95% CI		RERI	AP	SI
	Occurrence	Nonoccurrence		(Lower)	(Upper)			
(Hcy/HDL‐C)/(hs‐CRP/Alb)	70	134	–	–	–	6.407	53.82%	2.424
−/−	7	50	1.000	–	–	–	–	–
+/−	17	29	4.187	1.553	11.291	–	–	–
−/+	11	34	2.311	0.814	6.558	–	–	–
+/+	35	21	11.905	4.566	31.038	–	–	–

At 90 days, 21 patients experienced hemorrhagic transformation, yielding an overall incidence of 10.3%. This was significantly higher in the poor outcome group compared with the good outcome group (18.6% vs. 5.2%, *p* = 0.002). Elevated Hcy/HDL‐C was associated with a 2.1‐fold higher risk of hemorrhagic transformation (OR = 2.10, 95% CI: 1.30–3.50,*p* = 0.003). Moreover, elevated hs‐CRP/Alb was significantly correlated with higher all‐cause mortality at 90 days (6.6% vs. 0.9%; HR = 3.85, 95% CI: 1.12–13.22, *p* = 0.03).

Further classification of deaths within 90 days (*n* = 12) showed that eight cases (66.7%) were attributed to neurological causes, including cerebral herniation secondary to progression of large‐territory infarction (*n* = 4), severe neurological deterioration due to sICH (*n* = 3), and one case of systemic complication (multiple organ dysfunction) adjudicated on the basis of comprehensive medical record review as related to stroke/thrombolysis (*n* = 1). The remaining four deaths (33.3%) were due to non‐neurological causes, comprising pneumonia with respiratory failure (*n* = 2), acute myocardial infarction (*n* = 1), and malignant arrhythmia associated with electrolyte disturbance (*n* = 1).

Thus, the combined elevation of Hcy/HDL‐C and hs‐CRP/Alb not only amplified the risk of poor outcomes but was also strongly associated with hemorrhagic transformation and mortality, underscoring their potential as composite biomarkers for early clinical warnings.

### 3.5. Predictive Value of the Nomogram Model

A prognostic model incorporating admission NIHSS, atrial fibrillation, Hcy/HDL‐C, and hs‐CRP/Alb was developed based on multivariate regression. The model demonstrated excellent discrimination with a C‐index of 0.936 (95% CI: 0.904–0.968). ROC analysis yielded an AUC of 0.935, with a sensitivity of 87.1%, a specificity of 82.3%, and a Youden index of 0.694. Internal validation with bootstrap resampling produced a bias‐corrected C‐index of 0.931, a calibration slope of 0.978 (95% CI: 0.932–1.024), and no significant difference in the Hosmer–Lemeshow test (*χ* ^2^ = 7.32, *p* = 0.502), indicating good calibration (Figure 5A,B).

Figure 5Prognostic prediction model for ACI based on Hcy/HDL‐C and hs‐CRP/Alb ratios. (A) Decision curve of the prediction model; (B) calibration curve showing the relationship between predicted probabilities and observed outcomes; (C) nomogram of the prediction model.

**Figure:**
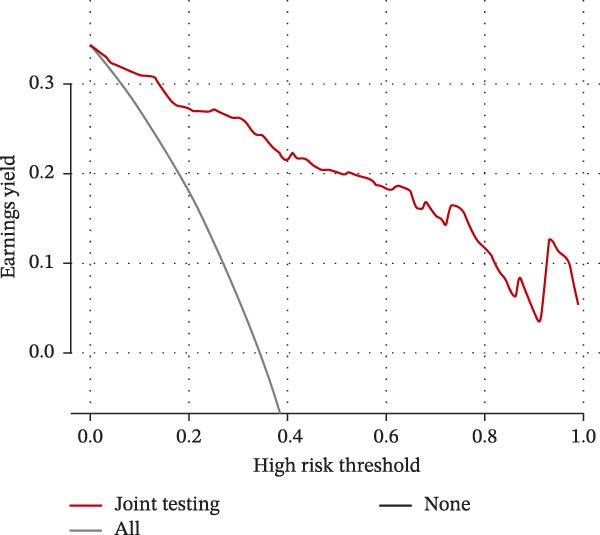
(A)

**Figure:**
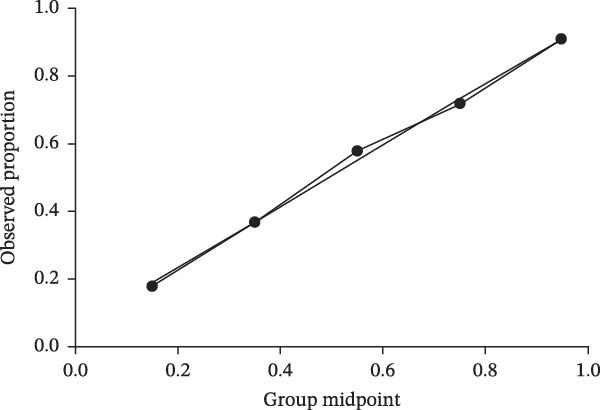
(B)

**Figure:**
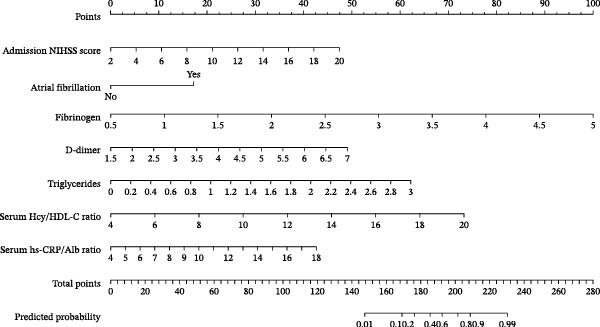
(C)

Compared with a traditional clinical model (NIHSS, atrial fibrillation, and fibrinogen), the new model demonstrated significantly improved predictive performance (C‐index 0.936 vs. 0.782, DeLong’s test *Z* = 5.17,*p*  < 0.001) (Figure 5C).

Collectively, the nomogram model based on Hcy/HDL‐C and hs‐CRP/Alb demonstrated superior discrimination and calibration compared to conventional clinical predictors, suggesting strong clinical utility for individualized risk stratification in ACI.

### 3.6. Subgroup Analyses

Subgroup analyses revealed heterogeneity across patient populations. In LAA subtype patients, Hcy/HDL‐C >10.62 × 10^−3^ was associated with a 3.2‐fold increased risk of poor outcomes (OR = 3.23, 95% CI: 1.48–7.01, *p* = 0.004). In CE subtype patients, hs‐CRP/Alb > 10.08 × 10^−5^ increased poor outcome risk by 2.7‐fold (OR = 2.71, 95% CI: 1.22–6.01, *p* = 0.012).

Among patients with severe strokes (NIHSS > 5), the combined predictive model achieved the highest performance, with an AUC of 0.942 (95% CI: 0.902–0.972), outperforming either biomarker alone (Table 5–Table 6).

**Table 5:** Subgroup analysis of predictive efficacy of Hcy/HDL‐C and hs‐CRP/Alb ratios.

Subgroups	Sample size (*n*)	Hcy/HDL‐C	*p*‐Value	hs‐CRP/Alb	*p*‐Value	*p*‐Value
		OR (95% CI)		OR (95% CI)		
Age						
≤65 years	112	1.83 (1.21–2.75)	0.004	2.14 (1.45–3.15)	0.001	—
>65 years	92	2.41 (1.82–3.21)	<0.001	1.97 (1.32–2.94)	0.007	**0.043**
Sex						
Male	126	2.05 (1.47–2.85)	<0.001	**3.12 (2.31–4.22)**	<0.001	**0.027**
Female	78	1.92 (1.21–3.05)	0.012	2.34 (1.58–3.47)	0.002	0.216
Hypertension						
Yes	154	2.18 (1.63–2.92)	<0.001	**2.76 (1.95–3.91)**	<0.001	**0.039**
No	50	1.64 (0.98–2.75)	0.058	1.89 (1.12–3.18)	0.032	0.352
Diabetes mellitus						
Yes	68	**2.89 (2.02–4.15)**	<0.001	2.25 (1.54–3.28)	0.001	**0.018**
No	136	1.95 (1.42–2.68)	<0.001	2.01 (1.42–2.84)	0.003	0.147

*Note:* OR and 95% CI: adjusted for baseline NIHSS score, atrial fibrillation, fibrinogen, D‐dimer, and triglycerides in multivariate logistic regression. Interaction *p*‐value: likelihood ratio test for heterogeneity across subgroups (e.g., >65 years vs. ≤65 years). Bold values: significant interaction (*p*  < 0.05) or enhanced predictive efficacy in the subgroup. Sample size indicates the number of patients with poor prognosis in each subgroup (total *n* = 70).

**Table 6:** Predictive performance of biomarkers stratified by TOAST classification and NIHSS scores.

Stratification variable	Hcy/HDL‐C OR (95% CI)	hs‐CRP/Alb OR (95% CI)	Interaction *p*‐value
TOAST classification			
‐ LAA (*n* = 112)	2.41 (1.78–3.27) ^∗∗^	1.89 (1.32–2.71) ^∗^	0.023
‐ SAO (*n* = 60)	1.45 (0.92–2.28)	1.21 (0.87–1.69)	0.312
‐ CE (*n* = 32)	1.78 (1.02–3.11) ^∗^	3.02 (2.15–4.24) ^∗∗^	0.008
NIHSS stratification			
‐ ≤5 points (*n* = 98)	1.92 (1.34–2.75) ^∗∗^	1.65 (1.18–2.31) ^∗∗^	0.165
‐ >5 points (*n* = 106)	2.31 (1.79–2.98) ^∗∗^	2.14 (1.68–2.73) ^∗∗^	0.087

*Note:* TOAST, Trial of Org 10,172 in Acute Stroke Treatment.

Abbreviations: CE, cardioembolism; CI, confidence interval; LAA, large artery atherosclerosis; NIHSS, National Institutes of Health Stroke Scale; OR, odds ratio; SAO, small artery occlusion.

^∗^ *p* < 0.05.

^∗∗^ *p* < 0.01.

Overall, Hcy/HDL‐C provided stronger predictive value in LAA patients, while hs‐CRP/Alb showed superiority in CE patients. The combination of both markers demonstrated optimal performance in severe cases, suggesting that risk interpretation should be tailored according to stroke subtype and disease severity.

## 4. Discussion

ACI remains a leading cause of death and disability worldwide. The prognostic value of inflammatory biomarkers and metabolism‐related indices has attracted increasing attention [18, 19]. In this study, we evaluated the roles of the Hcy/HDL‐C and hs‐CRP/Alb ratios in ACI prognosis after intravenous rt‐PA and developed a nomogram based on these two ratios. Both ratios were associated with poor outcomes, exhibited nonlinear dose–response relationships, and showed synergistic effects; the combined model demonstrated better discrimination and clinical utility than conventional clinical models, supporting its use for early risk stratification after thrombolysis.

Prior studies have largely focused on single biomarkers, yet their independent effects often attenuate or become unstable after multivariable adjustment [7]. Large‐scale studies [20, 21] indicate that composite indices integrating inflammation, metabolism, and nutritional status can improve predictive stability and clinical applicability. Our findings are consistent with this evidence: in multivariable models, Hcy/HDL‐C and hs‐CRP/Alb captured the overall state of inflammation–metabolism–nutrition imbalance more effectively than individual components; RCS and interaction analyses further revealed nonlinear features and synergistic effects between the two ratios. In thrombolysis cohorts, the CRP/Alb ratio has been reported to be independently associated with hemorrhagic transformation and poor functional outcomes [22], and a meta‐analysis also supports its association with increased risks of poor functional outcomes and mortality [23]. These data align with the direction of association observed for hs‐CRP/Alb with poor outcomes and safety endpoints in our study; our work further jointly modeled hs‐CRP/Alb and Hcy/HDL‐C and validated their interaction and nonlinear characteristics, extending the evidence base for composite ratios in postthrombolysis risk stratification.

Beyond serological ratios, our study indicates phenotypic heterogeneity in AF‐associated risk: among patients with AF, poor outcomes were associated with a higher prevalence of persistent/permanent AF and more frequent coexisting CAD and/or HF [24]. A stroke registry study systematically characterized the spectrum of cardiac disorders underlying ischemic cardioembolic events [25], showing that AF frequently coexists with structural cardiac disease and constitutes an important embolic substrate; in our rt‐PA‐treated population, linking AF phenotype and key cardiac comorbidities to functional outcomes provides additional evidence to support more granular stratification during the perithrombolysis period.

Mechanistically, in the thrombolysis setting, inflammation‐mediated blood–brain barrier injury represents an upstream pathway for hemorrhagic transformation; a thrombolysis cohort study showed that inflammation–nutrition imbalance reflected by elevated CRP and reduced albumin was associated with hemorrhagic transformation and poor functional outcomes [22]. An elevated Hcy/HDL‐C ratio indicates a relatively increased proatherogenic burden with insufficient HDL‐C protection, reflecting vascular dysfunction and metabolic homeostasis disruption [26, 27], whereas an elevated hs‐CRP/Alb ratio indicates an enhanced inflammatory response accompanied by reduced nutritional reserves [9, 11]. Together with the nonlinear and synergistic patterns observed in our study, these findings support a model in which heightened inflammation promotes endothelial injury and blood–brain barrier disruption, metabolic dysregulation exacerbates ischemic penumbral damage, and nutritional depletion limits repair and immune homeostasis, with convergent pathways contributing to increased risk of poor outcomes after thrombolysis.

This study has translational implications. In the emergency setting, combined measurement of Hcy/HDL‐C and hs‐CRP/Alb may facilitate identification of high‐risk patients and optimization of monitoring resources; during early postthrombolysis management, patients with concomitantly elevated ratios may warrant intensified control of inflammation and metabolic risk to reduce secondary injury and HT risk. The nomogram is visual and practical, enabling bedside individualized risk estimation and informing secondary prevention strategies.

Several limitations should be noted. First, this was a single‐center retrospective study; regional practice patterns and perithrombolysis management strategies may affect generalizability, and multicentre prospective cohorts are required for external validation and model calibration. Second, imaging quantitative measures and more granular phenotyping, such as NIHSS item scores, were not included, which may limit interpretability and transportability. Third, patients aged ≥85 years accounted for a small proportion of the cohort (*n* = 18, 8.8%), precluding an independent performance evaluation in this subgroup; given differences in comorbidity burden, stroke subtype distribution, and inpatient complication profiles in very old patients and evidence of higher in‐hospital mortality and complication‐related event burden [28], our findings should be further validated in cohorts aged ≥85 years, particularly among those treated with intravenous thrombolysis.

Further work should extend these findings in three directions. Longitudinal studies should characterize postthrombolysis trajectories of Hcy/HDL‐C and hs‐CRP/Alb and quantify their time‐dependent prediction of functional outcomes and thrombolysis‐related safety endpoints, with stratified external validation and recalibration of thresholds/models in key comorbidity groups (e.g., diabetes and CKD) and very old patients. Mechanistic studies should define how inflammation–metabolism–nutrition imbalance drives endothelial injury, blood–brain barrier disruption, and secondary brain injury and assess whether integrating multiomics data improves biological interpretability and prognostic performance. Prospective cohorts and randomized trials should then evaluate whether risk‐stratification‐guided integrated management targeting inflammation, metabolic risk, and nutritional status improves outcomes and reduces adverse events.

## 5. Conclusion

In summary, this study is the first to demonstrate the independent and synergistic prognostic value of Hcy/HDL‐C and hs‐CRP/Alb ratios in patients with ACI treated with intravenous thrombolysis. Their combined use not only provides sensitive and reproducible biomarkers for early identification of high‐risk patients but also offers novel insights for individualized interventions. Future multicenter, prospective, and multiomics studies are needed to validate and extend these findings, ultimately advancing precision management strategies for stroke patients.

**Nomenclature**
ACI: Acute cerebral infarction
AP: Attributable proportion
CE: Cardioembolic
CI: Confidence interval
C‐index: Concordance index
DCA: Decision curve analysis
HDL‐C: High‐density lipoprotein cholesterol
Hcy: Homocysteine
Hcy/HDL‐C: Serum homocysteine/high‐density lipoprotein cholesterol
hs‐CRP: High‐sensitivity C‐reactive protein
hs‐CRP/Alb: High‐sensitivity C‐reactive protein/albumin
LAA: Large‐artery atherosclerosis
LDL‐C: Low‐density lipoprotein cholesterol
mRS: Modified Rankin scale
NIHSS: National Institutes of Health Stroke Scale
ORs: Odds ratios
RCS: Restricted cubic spline
RERI: Relative excess risk due to interaction
rt‐PA: Recombinant tissue plasminogen activator
sICH: Symptomatic intracerebral hemorrhage
SI: Synergy index.

## Author Contributions

Mei Ding and Jie Dai contributed equally to the study design, data collection, and statistical analysis. Mei Ding drafted the initial manuscript. Jie Dai and Zhiyong Cao performed data interpretation and contributed to the construction and validation of the nomogram. Han Wang was responsible for patient data acquisition and follow‐up assessment. Xiangyang Zhu conceived and supervised the study, critically revised the manuscript for important intellectual content, and approved the final version for publication.

## Funding

This study was supported by the Scientific Research Project of Nantong Municipal Health Commission (Grant MS22022121).

## Disclosure

Xiangyang Zhu approved the final version for publication. All authors read and approved the final manuscript and agree to be accountable for all aspects of the work.

## Ethics Statement

This study was approved by the Clinical Ethics Committee of Nantong First People’s Hospital (Number 2022KT134).

## Consent

All the patients have been informed and signed informed consent before the experiments.

## Conflicts of Interest

The authors declare no conflicts of interest.

## Supporting Information

Additional supporting information can be found online in the Supporting Information section.

## Supporting information


**Supporting Information 1** Figure S1. Workflow of laboratory testing and ratio calculation. Note: This figure outlines the process of blood sample collection, separation and processing (blood cell count, coagulation function, serum biochemistry), testing methods, and calculation of key ratios (Hcy/HDL‐C and hs‐CRP/Alb).

**Supporting Information 2** Figure S2. Workflow of subgroup and sensitivity analyses. Note: The figure illustrates the stratification criteria (demographic characteristics, comorbidities, stroke subtype, and severity) and sensitivity analyses (exclusion of extreme values, multiple imputation, and threshold validation), to demonstrate the logic of model robustness testing.

## Data Availability

The data that support the findings of this study are available from the corresponding author upon reasonable request.
