# Supplementary figures and images for: Integrating Inflammation and Lipid Metabolism Biomarkers for Early Risk Stratification in Acute Cerebral Infarction: A Nomogram‐Based Approach
Source: Mediators Inflamm. 2026 May 7;2026:9960889. doi: 10.1155/mi/9960889 (PMC13150976; doi:10.1155/mi/9960889)

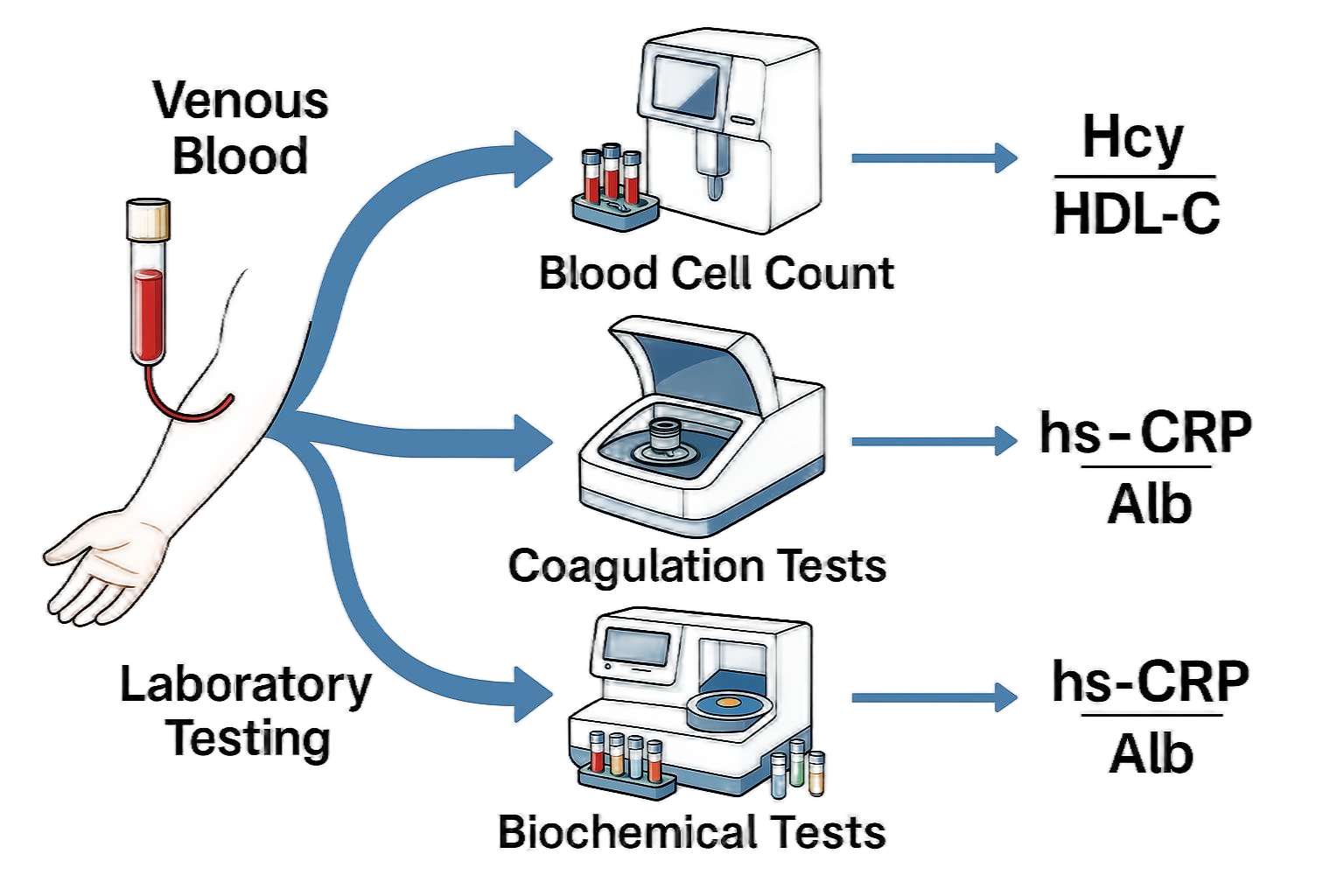

Supplement: Supplementary file 1 — Supporting Information 1 Figure S1. Workflow of laboratory testing and ratio calculation. Note: This figure outlines the process of blood sample collection, separation and processing (blood cell count, coagulation function, serum biochemistry), testing methods, and calculation of key ratios (Hcy/HDL‐C and hs‐CRP/Alb). [file MI-2026-9960889-s001.png]

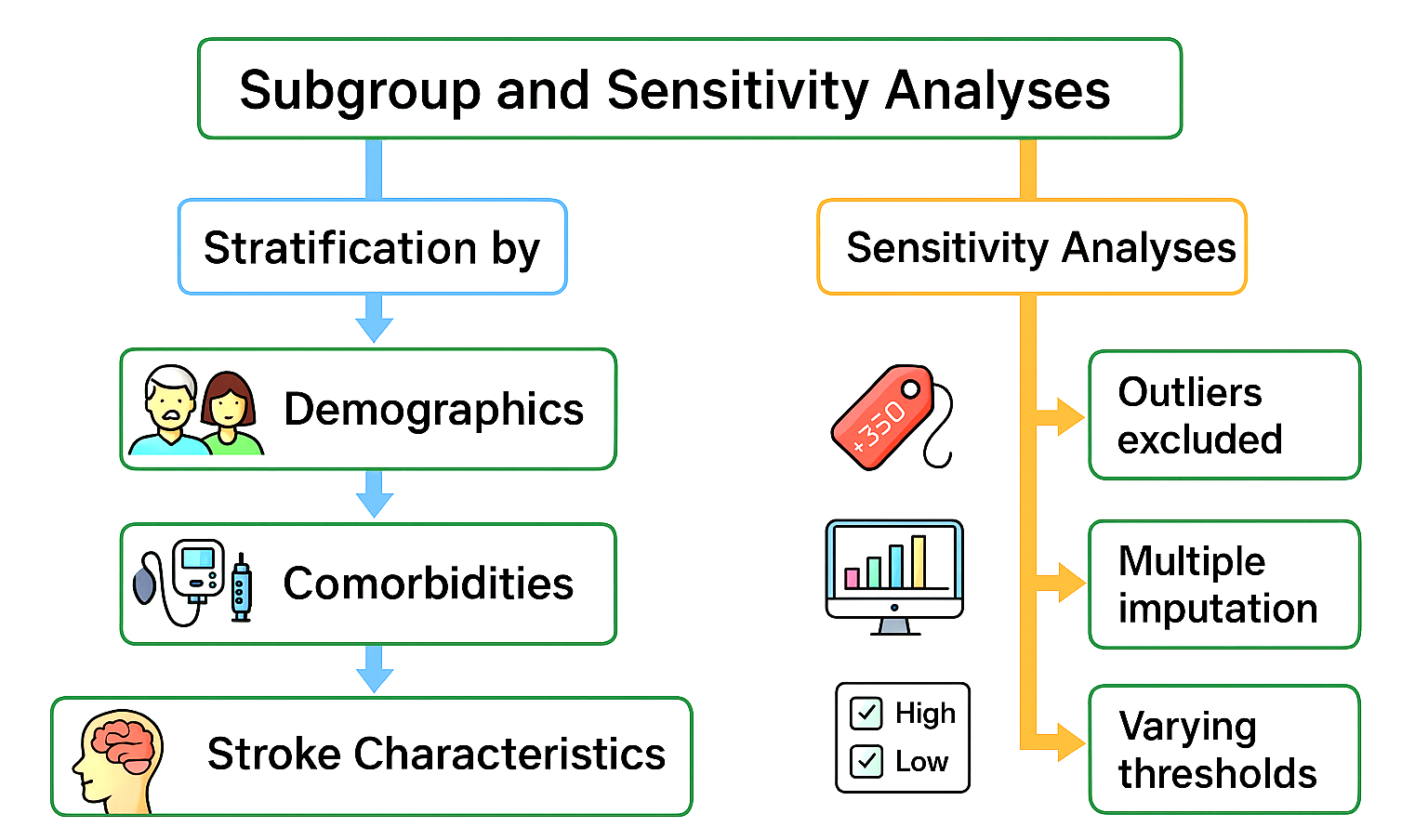

Supplement: Supplementary file 2 — Supporting Information 2 Figure S2. Workflow of subgroup and sensitivity analyses. Note: The figure illustrates the stratification criteria (demographic characteristics, comorbidities, stroke subtype, and severity) and sensitivity analyses (exclusion of extreme values, multiple imputation, and threshold validation), to demonstrate the logic of model robustness testing. [file MI-2026-9960889-s002.png]
